# Dataset of transcriptional landscape of B cell early activation

**DOI:** 10.1016/j.gdata.2015.06.007

**Published:** 2015-06-12

**Authors:** Alexander S. Garruss, Trent Fowler

**Affiliations:** a Wyss Institute for Biologically Inspired Engineering, Harvard University, Boston, MA 02115, USA; b Department of Genetics, Harvard Medical School, Boston, MA 02115, USA; c Department of Developmental, Chemical and Molecular Biology, Sackler School of Biomedical Science, Tufts University School of Medicine, 150 Harrison Avenue, Boston, MA 02111, USA

**Keywords:** Transcriptional, Landscape, B cell, Activation

## Abstract

Signaling via B cell receptors (BCR) and Toll-like receptors (TLRs) result in activation of B cells with distinct physiological outcomes, but transcriptional regulatory mechanisms that drive activation and distinguish these pathways remain unknown. At early time points after BCR and TLR ligand exposure, 0.5 and 2 h, RNA-seq was performed allowing observations on rapid transcriptional changes. At 2 h, ChIP-seq was performed to allow observations on important regulatory mechanisms potentially driving transcriptional change. The dataset includes RNA-seq, ChIP-seq of control (Input), RNA Pol II, H3K4me3, H3K27me3, and a separate RNA-seq for miRNA expression, which can be found at Gene Expression Omnibus Dataset GSE61608. Here, we provide details on the experimental and analysis methods used to obtain and analyze this dataset and to examine the transcriptional landscape of B cell early activation.

**Table:** 

Specifications	
Organism/cell line/tissue	*Mus musculus* C57BL6/Spleen/resting B cells
Sex	Male
Sequencer or array type	Illumina HiSeq 2000 and Genome Analyzer
Data format	Raw and analyzed
Experimental factors	Unstimulated, stimulated with anti-IgM or LPS
Experimental features	RNA-seq, ChIP-seq
Consent	Not applicable
Sample source location	Jackson Immunology, Bar Harbor, Maine, USA

## 1 Direct link to deposited data

http://www.ncbi.nlm.nih.gov/geo/query/acc.cgi?acc=GSE61608.

## 2 Experimental design

Experiments were designed to capture the earliest time point of a recognizably coordinated change in RNA expression of murine resting splenic B cells. A “coordinated change” was defined by changes in gene expression that correlated with pathways relevant to the stimuli; BCR signaling for BCR stimulus and NFKB/TLR for LPS stimulus, and was found lacking at 0.5 h but significant at 2 h stimulation [1]. At this earliest time point, we captured regulatory element associated sequences (ChIP-seq) to describe the regulatory landscape.

## 3 Materials and methods

### 3.1 Cells and induction

Naïve resting splenic B cells from 8 week old male C57BL6 mice (Jackson Laboratory, Bar Harbor, ME, USA) were isolated with anti-CD43 beads (Miltenyi), and confirmed as ~ 95% CD19 + by flow cytometry (FACSCalibur). The cells were re-suspended in ice-cold media (RPMI 1640 GlutaMAX with 25 mM HEPES (Life Technologies)/10% Gemini FetalPlex sera/55 nM B-mercaptoethanol (Life Technologies)) with either 10 μg/ml anti-mouse IgM goat IgG Fab fragments (Jackson Immunology) or 25 μg/ml *Salmonella* typhimurium typhus LPS (Sigma)). Afterwards, it was necessary to rest the cells on ice for 30 min prior to removing to an incubator at 37 °C/5% CO_2_ for the experimental times. Otherwise, we found that sera stimulation obscured specific stimulus stimulation. Monitoring expression of the ratio of either the primary response gene c-fos or the early activation marker CD69 over Actin B by RT-PCR offers a sensitive indicator of the resting state: both will be several folds above 1 over the resting state if activation occurs. Note that resting B cells have very low amounts of RNA; 25 million were used for each RNA-seq time point, while 10 million were sufficient for ChIP-seq. Animal care and use in this study are covered under the “Assurance of Compliance with PHS Policy on Humane Care and Use of Laboratory animals by Awardee Institutions” and approved by the Institutional Animal Care and Use Committee (IACUC) (Animal Welfare Assurance Number A-3775-01).

### 3.2 Deep sequencing

RNA libraries were prepared with the NuGEN Ovation RNA-Seq v2 library preparation kit, which amplifies both mRNA and non-polyadenylated transcripts. The resulting cDNA was fractionated by sonication and prepared using the Illumina TruSeq library preparation kit which was then multiplexed across single-end, 100 bp (initial RNA), 50 bp (and miRNA), lanes of an Illumina HiSeq 2000 sequencer using the Illumina pipeline RTA version 1.12.4.2 and de-multiplexing with CASAVA v1.8.2. Sequence data was aligned with Illumina ELAND software. Secondary RNA-seq and ChIP-seq for RNA Pol II was performed by the lab of Ali Shilatifard at the Stowers Institute using typical protocols with the Santa Cruz antibody N-20, sc-816x and chip libraries prepared with a KAPA Biosystems BHTP kit. H3K4me3 (Abcam antibody ab8580) and H3K27me3 (Abcam antibody ab6002) ChIP-seq was performed in the lab of Ranjan Sen at NIH using an Illumina TruSeq ChIP library kit according to the manufacturer's protocol followed by cluster generation with a TruSeq Cluster generation kit v5 and libraries were sequenced on an Illumina Genome Analyzer (GA-II) using Illumina RTA version 1.8.

### 3.3 Read mapping

Fastqc profiles showed good quality data (http://www.bioinformatics.babraham.ac.uk/projects/fastqc/). Non-manipulated reads were mapped against the mm9/ENSEMBL build 67 genome reference [2] using Tophat v2.0.10 [3] along with Samtools v0.1.19 [4] with general settings for RNA and bowtie 1.0.0 [5] for ChIP (options -n 1 -m 1 --best --strata). Mapped read numbers per million: RNA initial: rest 75.3, BCR30 20.9, BCR120 73.6, LPS30 44.7, and LPS120 40.5; RNA secondary: rest 77, BCR120 61, and LPS120 68.9; RNA Pol II: rest 18.1, BCR120 14.2, and LPS120 21.3; H3K4me3: rest 13.1, BCR120 16.4, LPS120 19.1; and K3K27me3: rest 19.0, BCR120 20.0, and LPS120 18.1.

### 3.4 Differential expression (DE) analysis

DE was identified by a minimal two-fold difference in log ratios of normalized reads generated with Cufflinks v1.3.1 using default settings [6]. Preferentially induced or reduced gene sets included genes that were identified by a change in either; a single response, or when affected by both responses with the preferred response changed at a ratio at least 2 fold more than the other non-preferred response. An XLSX spreadsheet of these results has been made available in the GSE61608 dataset.

### 3.5 MiRNA-seq analysis

After TRIzol isolation of RNA, Illumina's TruSeq Small RNA Sample Preparation Kits were used to produce material for generating 50 bp single end reads which were then analyzed with miRdeep2 [7] using the miRBase reference v14 with standard settings. Mapped miRNAs were confirmed by visual inspection of miRNA structure output from mirDeep2 and UCSC Genome Browser tracks [8], and inclusion in the Ensembl 67 data base [2]. Differential expression from the resting state was identified by a minimal two fold difference in miRdeep2 normalized reads. An XSLX spreadsheet of this analysis can be found in the GEO data set. Total miRNA dataset reads per million are the following: rest 20.2, BCR120 28.6, and LPS120 14.8. Total miRdeep2 miRNA reads (per thousand) are the following: rest 55.8, BCR120 16.2, and LPS120 27.6.

### 3.6 ChIP-seq analysis

To determine transcription start site (TSS) coverage, bedtools' “bamToBed” function and custom R scripts (bed2cov.R then cov2rpm.R) were used with R-2.11.1 to produce bedgraphs from bowtie mapped bam files. BedGraph file formats were then converted to BigWig files with bedgraphToBigwig for UCSC Genome Browser visualization (https://genome.ucsc.edu/goldenPath/help/bigWig.html). Reads-per-million-normalized coverage was computed for the gene sets and regions indicated with summary statistics, such as mean coverage, calculated at single base pair resolution. Histogram figures were made by converting the “cov2rpm.R” output using “R.prep_tss_matrix” followed by plot generation using “R.plot_tss_matrix”. The resulting TSS matrix is organized with transcript or gene names as the rows whereby the columns are positions flanking the start site of each transcript/gene. The matrix can easily be subsetted on transcript/gene names of interest, such as those found to be differentially expressed by another assay. Total normalized read coverage across other regions was created using the script “R.calculate_sums_and_max” and shown as boxplots in a manner similar to that shown in “R.plot_tss_matrix”. Standard R Wilcoxon Rank Sums Testing commands were used to determine the significance of differentially covered regions between biological samples. We have provided these scripts for reference. Note that cov2rpm.R and R.prep_tss_matrix require R-2.11.1 (http://cran.r-project.org/).

### 3.7 Quantitative PCR-RNA validation

Real-time PCR was performed with specific primers. Primer sequences have been made available in Supplement data from an earlier publication of the analysis of this dataset [1]. Target sequences are reported relative to Beta Actin and normalized to resting cells.
